# A Global Account of Established Non‐Native Fish Species

**DOI:** 10.1111/gcb.70451

**Published:** 2025-08-25

**Authors:** Phillip J. Haubrock, Mariana Novello, Neil Angelo Abreo, Dagmara Błońska, Ana Clara Sampaio Franco, Ismael Soto, Giuseppe Castaldelli, Stelios Katsanevakis, Antonín Kouba, Paride Balzani, Irmak Kurtul, Ali Serhan Tarkan, Elizabeta Briski, Robert Britton

**Affiliations:** ^1^ Department of Life and Environmental Sciences, Faculty of Science and Technology Bournemouth University Poole, Dorset UK; ^2^ Faculty of Fisheries and Protection of Waters University of South Bohemia in České Budějovice Vodňany Czech Republic; ^3^ CAMB, Center for Applied Mathematics and Bioinformatics Gulf University for Science and Technology Mubarak Al‐Abdullah Kuwait; ^4^ Graduate Program in Ecology (PPGE) Federal University of Rio de Janeiro Rio de Janeiro Brazil; ^5^ Mapua Malayan Colleges Mindanao Davao Philippines; ^6^ Department of Ecology and Vertebrate Zoology, Faculty of Biology and Environmental Protection University of Lodz Lodz Poland; ^7^ GRECO, Institute of Aquatic Ecology University of Girona Girona Catalonia Spain; ^8^ Department of Environmental and Prevention Sciences University of Ferrara Ferrara Italy; ^9^ Department of Marine Sciences University of the Aegean Mytilene Greece; ^10^ Marine and Inland Waters Sciences and Technology Department, Faculty of Fisheries Ege University Bornova, İzmir Türkiye; ^11^ Department of Basic Sciences, Faculty of Fisheries Muğla Sıtkı Koçman University Muğla Türkiye; ^12^ GEOMAR Helmholtz‐Zentrum für Ozeanforschung Kiel Kiel Germany

**Keywords:** aquatic ecosystem, aquatic habitat, biological invasion, freshwater ecosystem, introduction pathways, invasive species

## Abstract

The introduction of non‐native aquatic species has fundamentally transformed aquatic assemblages, primarily due to human activities, such as aquaculture, fisheries enhancement, aquarium trade, the creation of artificial corridors, and deliberate and accidental releases. Despite growing concern for biological invasions, there is no overall global appraisal of successful non‐native fishes. This study compiled a comprehensive dataset from several global sources to examine the taxonomic diversity, geographical distribution, introduction pathways, and ecological impacts of non‐native freshwater and marine fishes. Our dataset includes 1535 established non‐native fish species in 193 countries (82% of the global coverage), with Leuciscidae, Cichlidae, Salmonidae, and Cyprinidae being the most represented families. Although the incline in first reportings appears almost linear, annual reporting has been declining for decades, suggesting fish introduction rates are decreasing. The main introduction pathways are aquarium trade, aquaculture, fishery augmentation, and the creation of artificial corridors. The importance of introduction pathways substantially differed between freshwater species (primarily the aquarium trade and aquaculture) and marine species (corridors). While extensive records exist for hundreds of non‐native fish species, information on their impact types and impact mechanisms remains available only for a third of these species, highlighting broad knowledge deficiencies. Available impact information indicates that non‐native fish species may threaten native biodiversity through primarily competition and predation as dominant mechanisms. The magnitude of highest‐risk invasions suggests remediation is possible through urgent proactive policy and management interventions. This comprehensive global evaluation of established fish species and their ecological effects thus addresses critical data deficiencies, strengthens risk assessment frameworks, and supports the development of targeted biosecurity policies on priority pathways, approaches essential for helping mitigate the environmental and economic impacts of non‐native fish introductions.

## Graphical abstract

This study compiles the most complete global database of non‐native fish species that have successfully established themselves in new freshwater and marine environments. It documents 1535 species across 193 countries, identifies their main introduction routes—such as aquarium trade, aquaculture, and artificial waterways—and highlights that many threaten native biodiversity through competition and predation. The work reveals major knowledge gaps about impacts, especially in under‐studied regions, and calls for targeted management and biosecurity policies to reduce future invasions.

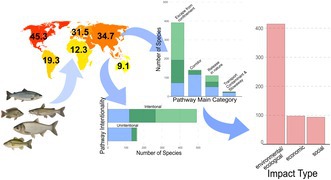

## 1 Introduction

Fish are among the most diverse and widely distributed vertebrate species globally (Rabosky 2020). Their global biodiversity has been shaped over millions of years by biogeographic patterns and climate variability that led to high species richness and endemism, with populations and communities playing critical roles in maintaining biodiversity, the structuring of food webs, and regulation of ecological processes (Albert et al. 2020; Su et al. 2021). Fish can provide multiple ecosystem services, including nutrient cycling, algae control, and the maintenance of trophic balances, increasing water quality and aquatic ecosystem stability (Lisbeth 2023). Fish also contribute to human well‐being, both directly and indirectly, by serving as an essential source of food and supporting food security and economic livelihoods for billions of people worldwide (Fiorella et al. 2014; Boyd et al. 2022), and presenting substantial cultural, recreational, and economic value (Embke et al. 2020; Shamsuzzaman et al. 2020).

Given the long historical relationship between fish and human societies, humans (e.g., during the expansions of the Roman Empire) have repeatedly facilitated the introduction of fish species beyond their native ranges for various purposes, including aquaculture, religious practices, biocontrol, and fisheries enhancement (Costa‐Pierce 2022). These introductions and translocations have contributed to the broad dispersal of numerous fish species, with many established populations (Kang et al. 2023; Tarkan, Kurtul, et al. 2024) contributing to the reshaping of entire biogeographic regions (Leroy et al. 2023). For example, common carp *Cyprinus carpio* is considered the oldest domesticated fish, with introductions dating back thousands of years (Nakajima et al. 2019). It is now regarded as one of the ‘worst’ non‐native species worldwide due to its substantial ecological impacts (Vilizzi 2012; Britton 2023). Among all non‐native taxa, introduced fishes are particularly notable for their high propagule pressure, where high numbers of individuals are often introduced simultaneously through multiple intentional introduction pathways, including aquaculture, ornamental trade, and angling, as well as accidental escapes (Carpio et al. 2019; Epa et al. 2023), and man‐made corridors, such as the Suez Canal (Katsanevakis et al. 2014; Balzani et al. 2022). This sustained and repeated introduction of fish species across different regions is exacerbated by the interconnected nature of freshwater ecosystems (e.g., human‐made canals linking river basins) that provide artificial water bodies for introductions and facilitate their subsequent spread across wide spatial areas (Leuven et al. 2009; Stringham and Lockwood 2021; Sandilyan 2024).

Aquatic ecosystems, especially freshwater, have been historically altered based on societal needs, with alterations such as impoundment making them particularly susceptible to fish invasions (Francis and Hardwick 2012; Marr et al. 2013). The ability of non‐native fish to survive, spread, and influence local ecosystems, however, depends on a complex interplay of biotic and abiotic factors (Liang et al. 2020; Milardi et al. 2022). Although not all non‐native fish species establish in their new environment, some can persist without reproducing (due to particular long life cycles or constant propagule pressure, e.g., *Oncorhynchus mykiss* ; Yoğurtçuoğlu et al. 2021), potentially exerting substantial ecological and socioeconomic impacts without expanding (Blackburn et al. 2011). Introduced fishes compete with native species, alter food web dynamics, disrupt habitat structures, and affect other fish populations through hybridization and disease transmission (Cucherousset and Olden 2011; Haubrock et al. 2022; Britton 2023). Competition between non‐native and native fish is often asymmetric due to non‐native individuals having higher foraging rates and/or more aggressive competitive behavior, enabling their greater resource acquisition, particularly in degraded ecosystems (Bergstrom and Mensinger 2009; Abrahams et al. 2017). Increased predation pressure by non‐native fish can drive biodiversity loss, as observed with the Nile perch *Lates niloticus* (Kaufman 1992) and the peacock bass *Cichla monoculus* (Sharpe et al. 2017). Additionally, their introduction can release novel diseases (including both pathogens and parasites; Ercan et al. 2015; Kuchta et al. 2018; Spikmans et al. 2020). Ecological engineering species can also alter habitat structure, further degrading aquatic ecosystems (Matsuzaki et al. 2009). The cumulative effects of these impacts can extend beyond ecological systems, imposing high economic burdens on fisheries, aquaculture, and water infrastructure, with global costs estimated at billions of dollars globally (Haubrock et al. 2022).

While existing studies have addressed various aspects of fish invasions (e.g., Dawson et al. 2017; Bernery et al. 2024), critical gaps that remain are comprehensive assessments that integrate multiple components of the invasion process, including holistically approaching (a) introduction pathways, (b) traits of non‐native species that contribute to successful establishment (but see Bernery et al. 2023), (c) the mechanisms and consequences of invasion impacts, and (d) effective management and mitigation strategies (Bernery et al. 2024). Given the notable impacts of established non‐native fish species globally, it is imperative to document their diversity, distribution, and invasion dynamics. A robust and comprehensive inventory of established non‐native fish is essential for informing management efforts and mitigating potential future invasions. To fill this critical gap, this study compiles the most comprehensive global assessment of established non‐native fish species yet, with a particular focus on introduction pathways and impacts. By synthesizing data on freshwater fish invasions at both global and continental scales, this work identifies patterns and knowledge gaps, while highlighting future research priorities and providing a more effective and evidence‐based understanding of biological invasions.

## 2 Methods

### 2.1 Composition and Distribution

To investigate established non‐native fish species (Actinopterygii) globally (defined as those non‐native species that reproduce ≥ *n* generations independently of new introductions in an area to which they are not native and have no evolutionary history; *sensu* Soto et al. 2024), we used a dataset recently published by Briski et al. (2024). This database, used in conjunction with the *Global Biodiversity Information Facility* (GBIF) (Svenningsen and Schigel 2024), integrates data from the SInAS (*Standardising and Integrating Alien Species*) workflow of non‐native species occurrences (Seebens et al. 2020, 2021) and presents, to our knowledge, the most up‐to‐date compilation of established non‐native fish species. Subsequently, species listed as “CASUAL” or “ABSENT” in the ‘degreeOfEstablishment’ (indicating the level of establishment, e.g., established or casual) and ‘occurrenceStatus’ columns (indicating whether the species is present or absent) in Seebens et al. (2021) were removed due to their ambiguous status following the improved Darwin Core for the research of non‐native species by Groom et al. (2019). The remaining species listed as “introduced” in column ‘EstablishmentMeans’ (referring to the species' respective status, i.e., native, introduced, nativeReintroduced) were either blank in column ‘degreeOfEstablishment’ or listed as ‘established’ (Supplement S1).

Verifying ‘establishment’ is often challenging since it pertains to the ‘population‐level’ (Haubrock et al. 2024), and multiple populations of the same species may be at different stages of the invasion process and can change dynamically over time (Soto et al. 2024). Therefore, we acknowledge the possibility that erroneous (or not updated) entries (i.e., erroneous classification of the degree of establishment) could have been transferred from the original sources (i.e., Seebens et al. 2017, 2021; Casties et al. 2016) to Briski et al. (2024) and might not have been caught during subsequent manual checking. The same might have occurred with valid species names, taxonomy, and species status. Thus, in the final step, species identities and scientific names were first manually verified against GBIF, Eschmeyer's catalog of fishes (Fricke et al. 2025), and *FishBase* (www.fishbase.com; Froese and Pauly 2024). If a species was not found in these databases, we conducted general internet searches in January 2025 to confirm its authenticity before correcting misspelled names and removing duplicate entries from the dataset. Then, we verified the occurrence of each species in every country listed in our dataset by cross‐referencing reported occurrences with GBIF data. If GBIF did not report the presence of a non‐native fish species in a country where it was listed in our dataset derived from Briski et al. (2024), we conducted manual checks using www.google.com and www.scholar.google.com. This process resulted in a final dataset comprising 1535 species and 5412 entries globally. However, to confirm observed patterns and trends in the full dataset, a sensitivity analysis was conducted by re‐analysing the conservative subset of the data encompassing only records listed as “established” in column ‘degreeOfEstablishment’; confirming both spatial patterns and trends (Supplement S2).

For every species reported in our dataset, the habitat it occupies was assigned (i.e., freshwater, marine, or both). One or more habitats were assigned for each species based on the Step2_StandardTerms_GRIIS file (Seebens et al. 2021), originating from the *Global Register of Introduced and Invasive* Species (GRIIS; Pagad et al. 2018). If habitat information was not automatically assigned to a species, we conducted an additional manual search using the *World Register of Marine Species* and a general internet search between July and September 2024 to classify the species (Briski et al. 2024). Brackish habitats were included in the marine habitat based on the Venice System (1958).

### 2.2 Distribution and Native Range

To explore the spatial variation underlying the distribution of established non‐native fish species, we first investigated the global distribution by country, including the average number of established non‐native fish species per country in each given continent. Then, we examined the cases of key countries where the largest recorded numbers of established non‐native fish species had been documented, namely the United States (USA) and Mexico in North America, Türkiye, Israel, China, the Philippines, Indonesia, and Japan in Asia, and Italy in Europe (see Figure 2). The distinguishing characteristics that set these countries apart include their surface area, geographical location, climate, and socio‐economic factors (Sadovníkova et al. 2021). To facilitate meaningful comparisons through data standardization, we expressed the number of reported species per unit area, using a standardized area unit of 10,000 km^2^.

The native range of each established non‐native fish species was identified using web‐scraping on several online sources, such as *FishBase*, *fishipedia* (www.fishipedia.es), and GBIF, combined with manual internet searches (all sources are listed in Supplement S1). Based on the identified native range of a species, the biogeographic division (realm) of the Earth (Nearctic, Neotropical, Palearctic, Afrotropical, Indo‐Malayan, Australasia, Oceania, and Antarctica) was assigned to all fish species, including marine species. However, the authors acknowledge that the classification from Spalding et al. (2007) is predominantly used for marine assessments. Given our assessment's cross‐ecosystem nature that included species occurring in both freshwater and marine ecosystems, the much broader biogeographic division from Dinerstein et al. (2017) was used. The Oceanian and Australasia realms were combined due to significant similarities in their species composition. Please also note that some species' native range encompasses multiple realms, which were then designated as such (e.g., Neotropical‐Afrotropical). Species reported in the SInAS database as non‐native to a specific country, but with unknown native regions (cryptogenic species), were kept in our analyses, while those cryptogenic species that are widely distributed across more than two biogeographic realms were considered cosmopolitan and thus removed from our dataset. Finally, if species originate from a different continent, they were always considered established non‐native; however, for species from the same continent, nativeness is acknowledged as a subject of ongoing debate, given that native ranges have shifted continuously over time and the boundaries are largely assumed (Oficialdegui et al. 2024).

### 2.3 Temporal Reporting

To understand the temporal dynamics of fish invasions globally, we extracted the first record of non‐native fish (i.e., the earliest record of a species in a given location) on the continent using the *Global Alien First Records Database* (v3.1. Seebens et al. 2017). We then estimated the cumulative incline of annual reporting per continent and the marginal incline in newly established non‐native fish species reported annually. Similar to the comparison of surface area standardized species accounts, we examined the temporal dynamics in the reporting of established non‐native fish species in China, Indonesia, Israel, Italy, Japan, Mexico, the Philippines, Türkiye, and the USA to identify variation in the temporal recording of these species.

### 2.4 Pathways of Introduction

To assess the introduction pathways of non‐native fish, we used the data from Saul et al. (2017), which follows the standardized categorization scheme recently adopted by the *Convention on Biological Diversity* (CBD; Tsiamis et al. 2017), integrating information from major invasive non‐native species databases, such as the *Global Invasive Species Database* (GISD; Poorter and Browne 2005) and DAISIE (Hulme et al. 2010). This dataset was further complemented by retrieving pathway information from the *European Alien Species Information Network* (EASIN; Katsanevakis et al. 2015). For each recorded pathway, information was structured into three key components: (i) the main pathway category (following the CBD scheme: Release in nature, Escape from confinement, Transport as contaminant, Transport as stowaway, and Corridors); (ii) a subcategory providing further detail on the specific mode of introduction (e.g., biological control, agriculture, ornamental trade, ballast water transport; Saul et al. 2017); and (iii) the intentionality of the introduction (i.e., whether the species was introduced deliberately or arrived unintentionally). This structured approach improves comparability across datasets and facilitates a more precise assessment of introduction mechanisms.

### 2.5 Impact Information

Acknowledging that the introduction of a non‐native species is always accompanied by a form of impact of varying magnitude (i.e., due to predation, competition, resource use, etc.; Soto et al. 2024), we individually extracted information if an impact was reported globally from the *Global Register of Introduced and Invasive Species* (GRIIS; Pagad et al. 2018) and the *Global Impacts Dataset of Invasive Alien Species* (GIDIAS; Bacher et al. 2025) for the established non‐native fish species in our dataset. Due to the limited information on types and mechanisms of impact reported in GRIIS and GIDIAS, we conducted a manual search for all established non‐native fish species in our dataset and searched for additional information in the *Centre for Agriculture and Bioscience International* (CABI Compendium; CABI 2025), the *rfishbase* R package, which is an interface to the www.fishbase.org database (Froese and Pauly 2024), and finally www.scholar.google.com, compiling the currently most up‐to‐date information on impacts (i.e., environmental/ecological, social, and economic) and [ecological] impact mechanisms (i.e., competition, disease transmission, herbivory/grazing, hybridization, interaction, predation, rapid [population] growth).

The final dataset, named the *Global Fish Invasion Database* (GFID), is freely accessible (https://github.com/IsmaSA/GFID), providing comprehensive information on species identities, native ranges, established non‐native occurrences by country, habitats, introduction pathways, and documented impacts, forming the most complete global resource on established non‐native fish invasions to date.

## 3 Results

### 3.1 Composition and Distribution

**FIGURE 1:**
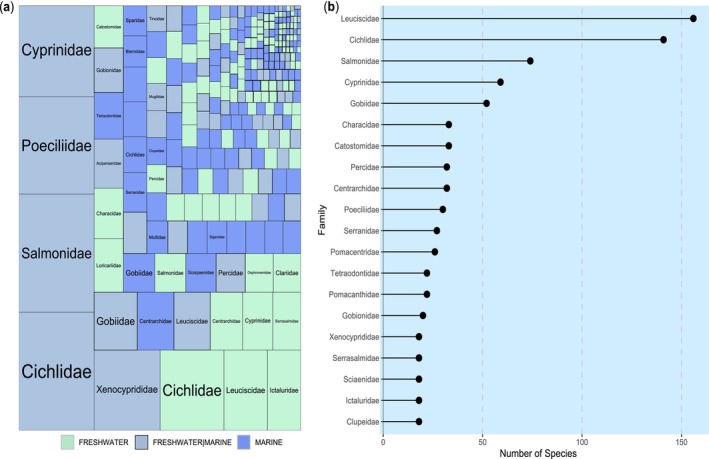
Treemap showing the distribution of total species richness among families of established non‐native fish by ecosystem (a) and the top‐20 most species‐rich families of established non‐native fish globally (b).

The data collated by Briski et al. (2024) contained information on *n* = 1535 established non‐native fish species globally, reported from *n* = 193 countries, spanning *n* = 204 families (Figure 1a; Supplement S1). Of these established non‐native fish species, *n* = 771 were classified as freshwater species, *n* = 451 as marine species, and *n* = 314 as occurring in both freshwater and marine environments, unevenly distributed across families (Table 1). The most species‐rich family was Leuciscidae (*n* = 156 species), followed by Cichlidae (*n* = 141), Salmonidae (*n* = 74), Cyprinidae (*n* = 59), and Gobiidae (*n* = 52), with all other families of < 50 species (Figure 1b). Regarding global occurrences, Cichlidae had the highest number of reports (*n* = 671), then Salmonidae (*n* = 455), Cyprinidae (*n* = 388), Poeciliidae (*n* = 372), Xenocyprididae (*n* = 257), Leuciscidae (*n* = 253), Centrarchidae (*n* = 225), Gobiidae (*n* = 176), and Ictaluridae (*n* = 125). All other families had < 100 country records overall.

**TABLE 1:** Top‐20 most species‐rich families of established non‐native fish species globally, broken down by number (#) and percentage (%) across Marine (M), Freshwater/Marine (FM), and Freshwater (F) species.

#	Family	Total	# M	% M	# FM	% FM	# F	% F
1	Leuciscidae	156	0	0	23	14.7	133	85.3
2	Cichlidae	141	7	5	29	20.6	105	74.5
3	Salmonidae	74	0	0	56	76	18	24
4	Cyprinidae	59	0	0	22	37.3	37	62.7
5	Gobiidae	52	28	53.8	20	38.5	4	7.7
6	Catostomidae	33	1	3	1	3	31	93.9
7	Characidae	33	0	0	0	0	33	100
8	Centrarchidae	32	4	12.5	1	3.1	27	84.4
9	Percidae	32	0	0	7	21.9	25	78.1
10	Poeciliidae	30	1	3.3	15	50	14	46.7
11	Serranidae	27	27	100	0	0	0	0
12	Pomacentridae	26	26	100	0	0	0	0
13	Pomacanthidae	22	22	100	0	0	0	0
14	Tetraodontidae	22	13	59.1	7	31.8	2	9.1
15	Gobionidae	20	0	0	4	20	16	80
16	Clupeidae	18	8	44.4	8	44.4	2	11.1
17	Ictaluridae	18	0	0	1	5.6	17	94.4
18	Sciaenidae	18	16	88.9	0	0	2	11.1
19	Serrasalmidae	18	1	5.6	0	0	17	94.4
20	Xenocyprididae	18	0	0	10	55.6	8	44.4

### 3.2 Distribution and Native Region

**FIGURE 2:**
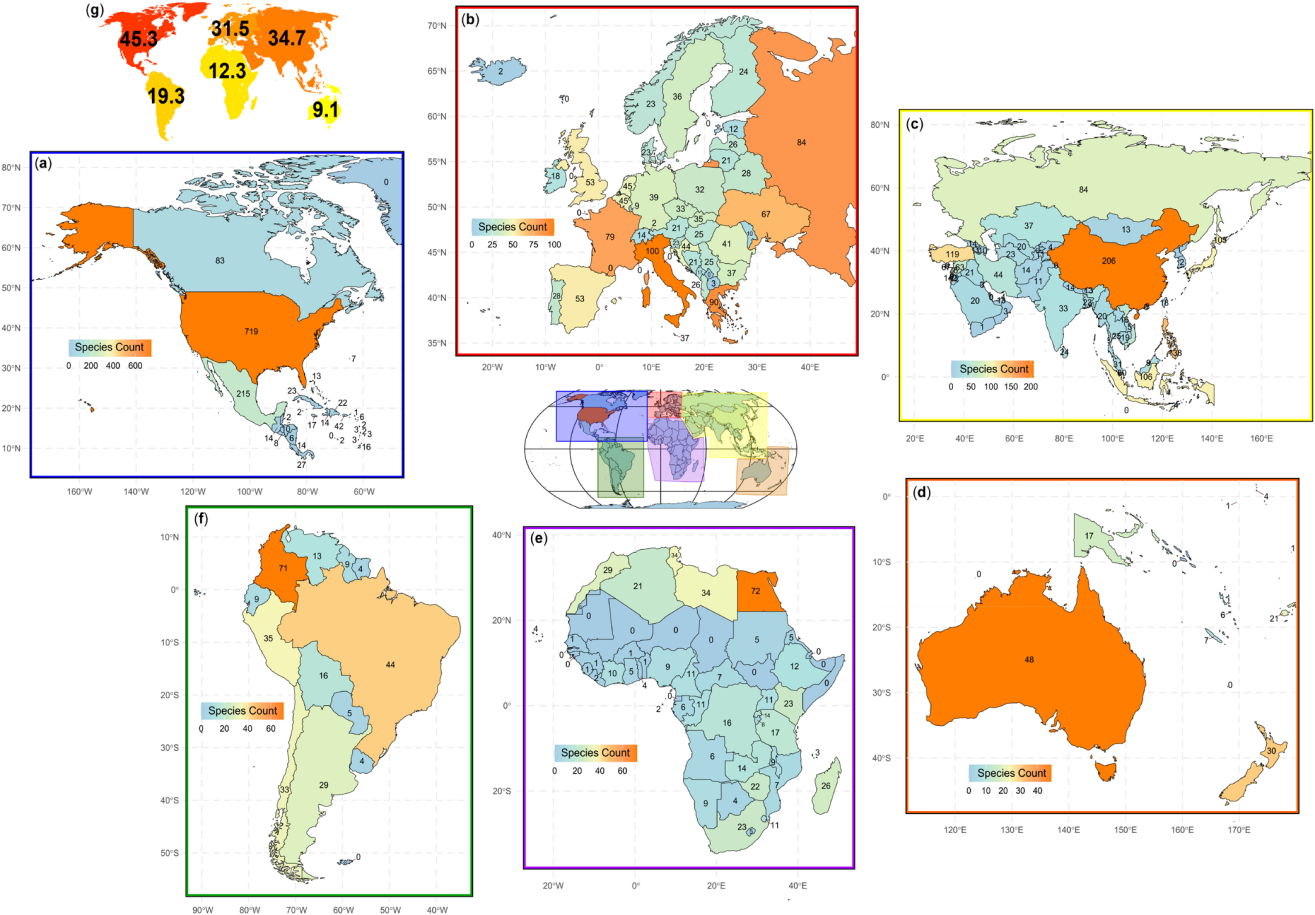
Global distribution of established non‐native species broken down by continent: (a) North America; (b) Europe; (c) Asia; (d) Oceania; (e) Africa; (f) South America; and the national average per continent (g). Note that the shading for each continent's countries is based on their own continent‐specific scaling. Map lines delineate study areas and do not necessarily depict accepted national boundaries.

The global distribution of established non‐native fish species was heterogeneous continentally, with North America having the highest number (*n* = 841), followed by Asia (*n* = 722), Europe (*n* = 363), Africa (*n* = 180), South America (*n* = 154), and finally Oceania (*n* = 91) (Figure 2). At the country level, the USA was the most species‐rich, with *n* = 719 established non‐native fish species reported, followed by Mexico (*n* = 215) and China (*n* = 206). Other countries with over 100 species included Israel (*n* = 142), the Philippines (*n* = 138), Türkiye (*n* = 119), Indonesia (*n* = 106), Japan (*n* = 105), and Italy (*n* = 100). For all other countries, less than 100 species were listed (Supplement S1). When averaged across each respective continent's countries, North America had the highest number of established non‐native fish species per country (*n* = 45.3), followed by Asia (*n* = 34.7) and Europe (*n* = 31.5). Relatively low mean species numbers were in South America (*n* = 19.3 species), Africa (*n* = 12.3), and Oceania (*n* = 9.1). Notably, data for some African countries were incomplete (Figure 2). After standardizing by surface area (species per 10,000 km^2^), the most invaded countries were Israel (6.5 species per 10,000 km^2^), followed by the Philippines (4.6 species per 10,000 km^2^), Italy (3.3 species per 10,000 km^2^), Japan (2.8 species per 10,000 km^2^), and Türkiye (1.5 species per 10,000 km^2^). Other notable countries included Mexico (1.1 species per 10,000 km^2^), the USA (0.8 species per 10,000 km^2^), Indonesia (0.6 species per 10,000 km^2^), and China (0.2 species per 10,000 km^2^).

**FIGURE 3:**
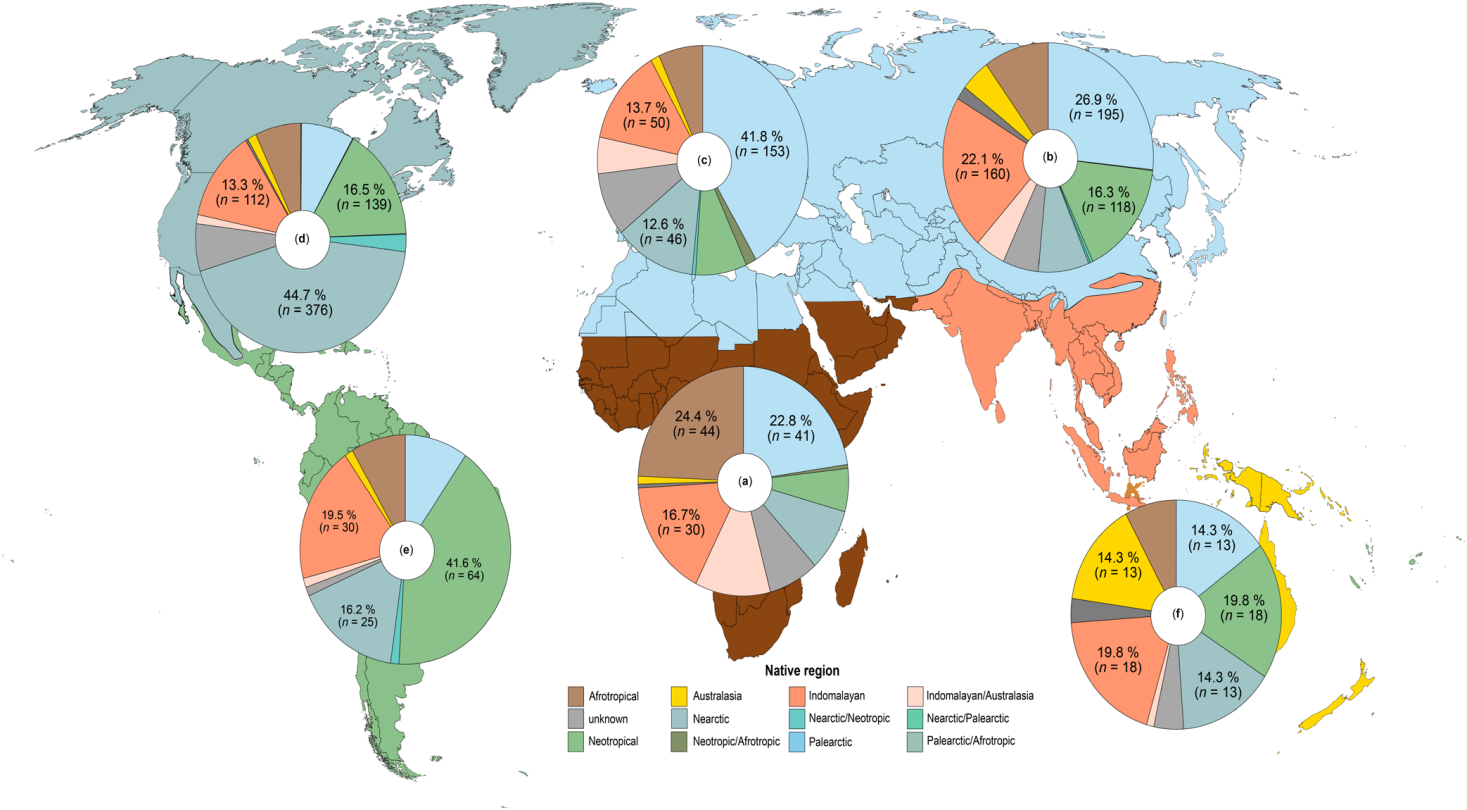
Native range of established non‐native species broken down by continent (Africa, Asia, Europe, North America, South America, and Oceania), indicating the percentage and number for the three main contributor biogeographical realms of established non‐native fish species. Map lines delineate study areas and do not necessarily depict accepted national boundaries.

The native range of established non‐native fish species differed markedly by continent, with distinct patterns of biogeographic contribution. The Palearctic realm contributed most species to Europe (41.6%) and Asia (26.7%), reflecting its influence in temperate regions. In North America, the Nearctic realm dominated (43.4% of species), while in South America, the Neotropical realm contributed the highest proportion (41.3%), consistent with its tropical and subtropical ecosystems. Africa displayed a balanced distribution, with the Afrotropical realm contributing the most species (24.4%), followed by the Palearctic (22.7%) and the Indomalaya (16.6%) realms. Aside from North America, Africa was the only continent where the majority of established non‐native species originated from realms within the continent itself due to 24.3% originating from the Afrotropical and 0.6% from Afrotropical‐associated (e.g., Neotropical‐Afrotropical, 0.6%) regions. In Oceania, contributions were more evenly distributed, with the Australasian and Neotropical realms, each representing 19.3% of the species, followed by the Palearctic (14.8%) and Nearctic (14.8%) realms. Notably, mixed realms (e.g., Indomalaya‐Australasian) also contributed significantly, particularly in regions with shared biogeographic histories like Oceania and Asia (Figure 3).

### 3.3 Temporal Reporting

**FIGURE 4:**
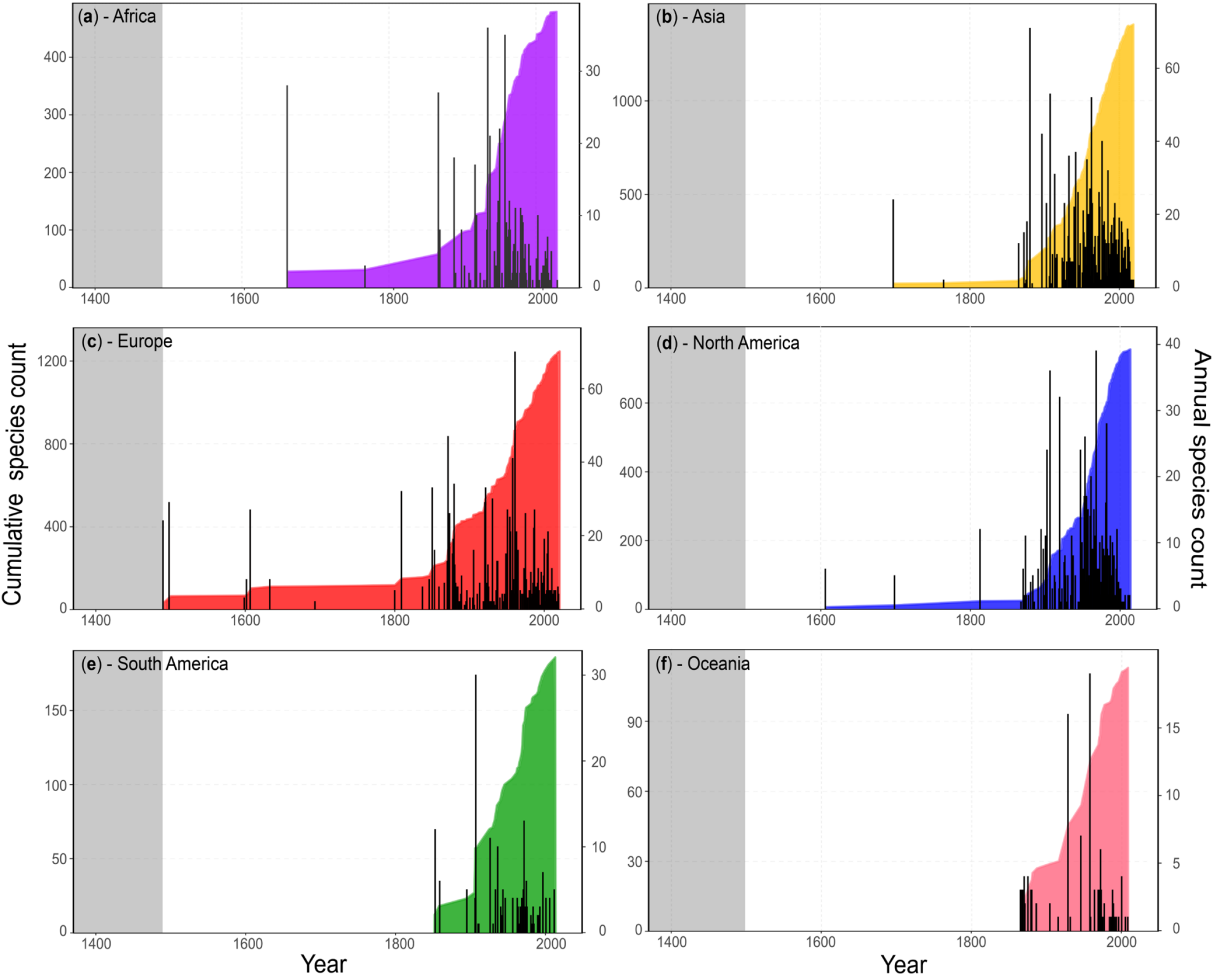
Cumulative (left *y*‐axis; coloured area) and marginal counts (right *y*‐axis; black bars) of established non‐native fish species reported for the first time per continent: (a) Africa, (b) Asia, (c) Europe, (d) North America, (e) South America, and (f) Oceania. The grey‐shaded area indicates the period before 1492, marking the onset of the Columbian Exchange, which can be considered as a milestone for global species introductions.

First record data was available for 1258 of 2368 entries (53%) of established non‐native species, including 119 of 180 established non‐native fish species in Africa, 433 of 722 in Asia, 294 of 368 in Europe, 296 of 841 in North America, 61 of 154 in South America, and 55 of 103 in Oceania. Cumulatively counting records of established non‐native fish species showed an overall increase across all continents, with a marked acceleration from the early to late 19th century; this was mirrored by a comparably abrupt appearance of high numbers of established non‐native fish species, which leveled off in successive years (Figure 4). A breakdown by country, for species for which the year of the first record was available, can be found in Supplement S3.

### 3.4 Pathways of Introduction

**FIGURE 5:**
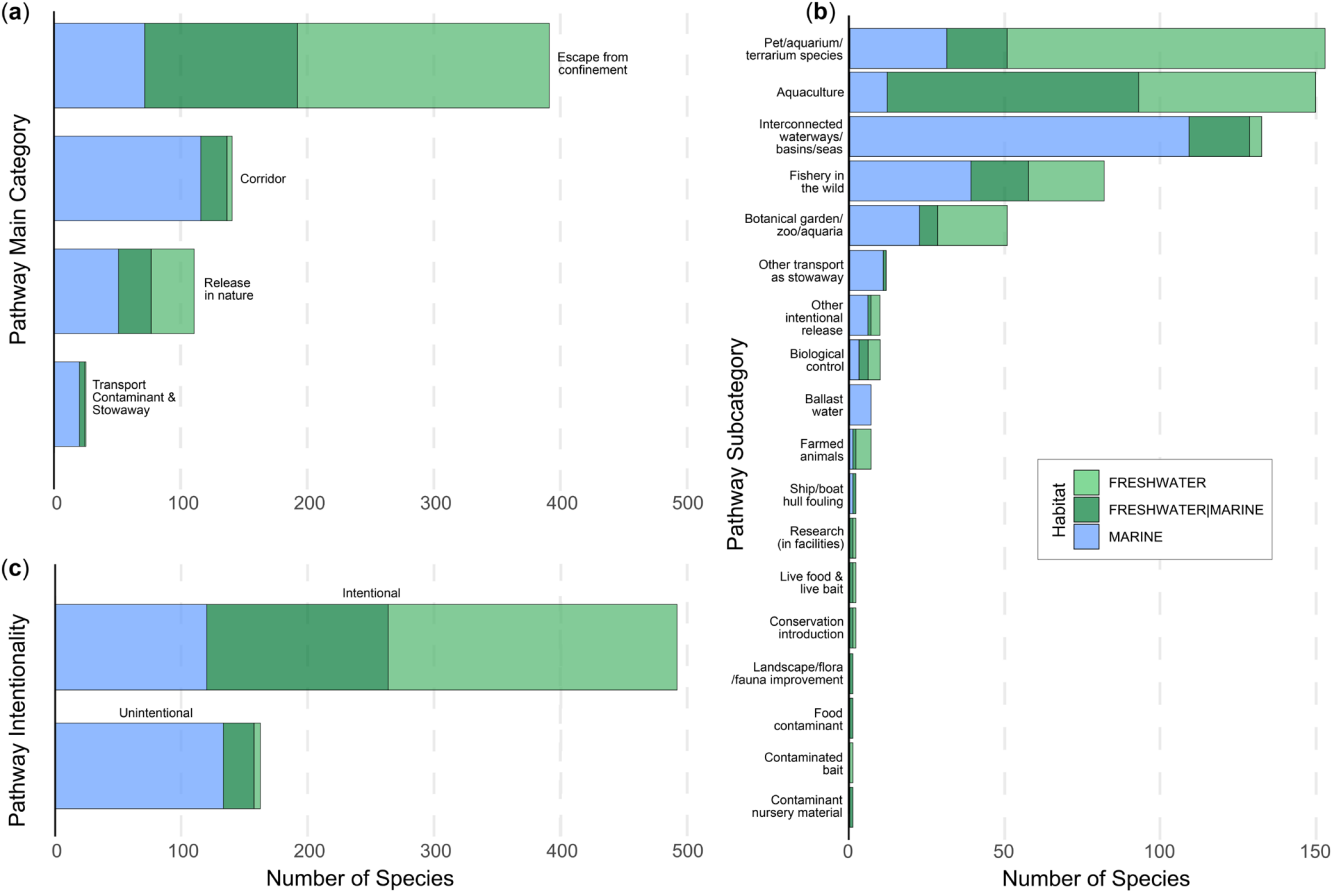
Main pathway categories (a) and subcategories (b) according to the CBD classification broken down by habitat, available for established non‐native fish species listed by Briski et al. (2024), as well as information on whether their introduction was intentional or unintentional according to Saul et al. (2017) (c). The grouping of these pathway subcategories under their respective main categories is provided in Supplement S4.

The main pathway was ‘escape from confinement’ with *n* = 379 listed established non‐native fish species, followed by ‘corridor’ (*n* = 136), ‘release in nature’ (*n* = 107), and finally ‘transport (contaminant and stowaway)’ (*n* = 14) (Figure 5a). The most frequent pathway subcategories were ‘pet/aquarium/terrarium species’ (*n* = 157) and ‘aquaculture’ (*n* = 150), followed by ‘interconnected waterways/basins/seas’ (*n* = 128), ‘fishery in the wild’ (*n* = 84), and ‘botanical garden/zoo/aquaria’ (*n* = 52). Other pathway subcategories encompassed less than 50 established non‐native fish species each (Figure 5b). Finally, introductions were classified as primarily ‘intentional’ (*n* = 486), while unintentional introductions were documented for *n* = 160 species (Figure 5c). The importance of pathways differed between marine and freshwater introductions, with corridors (interconnected waterways/basins/seas) being the most important pathway for marine fish, whereas escape from confinement (via the pet/aquarium/terrarium species trade or aquaculture) for freshwater fish. The patterns of pathway categories and subcategories were generally consistent across continents (Supplement S4).

### 3.5 Impacts

**FIGURE 6:**
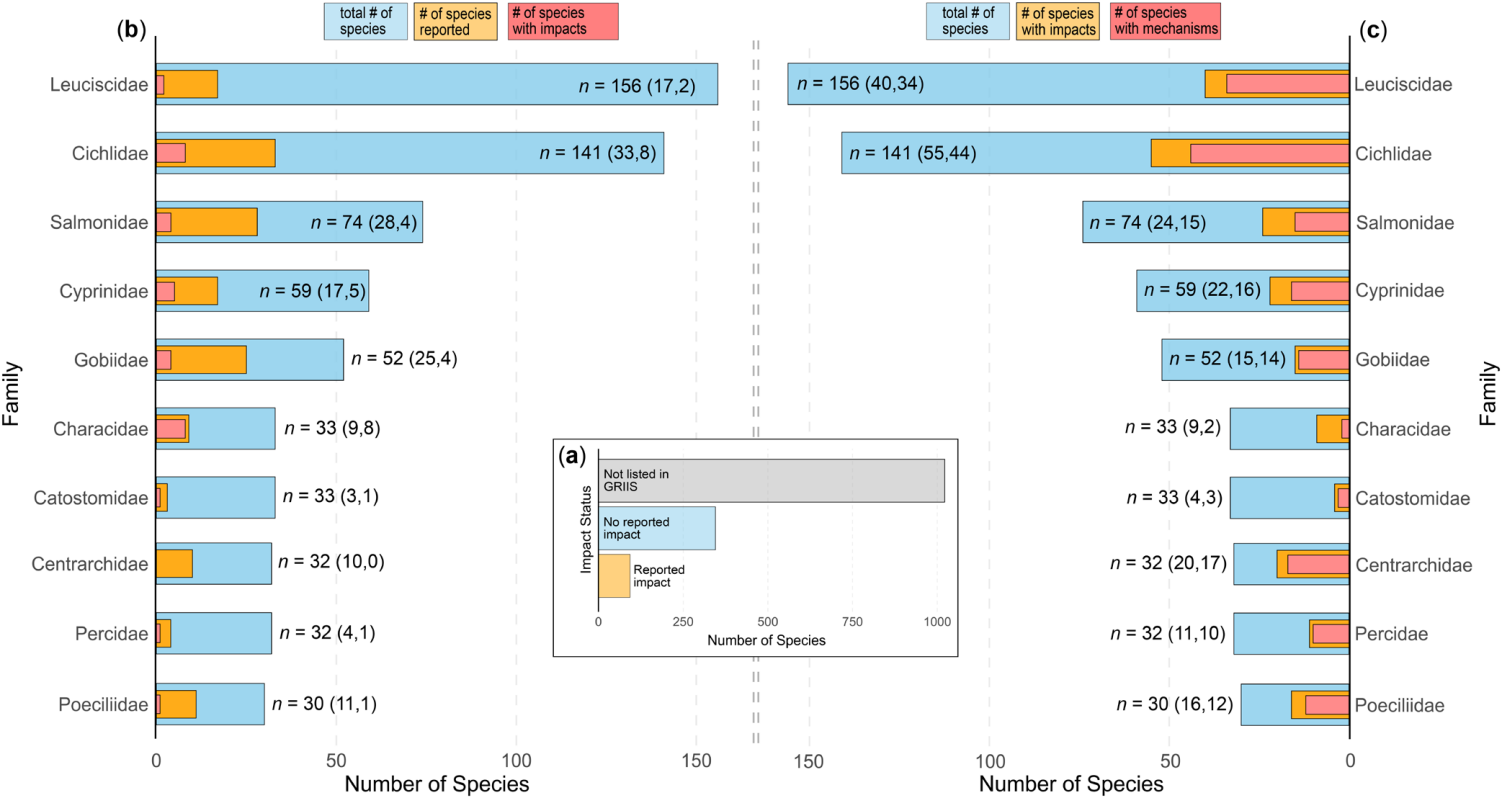
An overview of the overall numbers of fish species which had a reported impact, had no reported impacts, or species that were missing in GRIIS (a), the top‐10 of most species‐rich families of established non‐native fish species with their respective number of species for which impacts were reported in the Global Register of Introduced and Invasive Species (GRIIS) (b) and according to the data compiled in the Global Fish Invasion Database (GFID) (c).

A total of *n* = 455 species of established non‐native fish were listed in GRIIS, from which *n* = 80 had a detected impact and *n* = 375 had no reported impact. Information was missing for *n* = 1080 species (Figure 6a). Besides being the most species‐rich family, Leuciscidae was the family with only 10.9% (*n* = 17 of 156) reported in GRIIS and only 1.3% with reported impacts (*n*_impact_ = 2 of 156), followed by Cichlidae with 23.4% being reported (*n* = 33 of 141) and 5.7% having a reported impact (*n*_impact_ = 8 of 141), Salmonidae with 37.8% (*n*_impact_ = 28 of 74) and 5.4% (*n*_impact_ = 4 of 74), Cyprinidae with 28.8% (*n* = 17 of 59) and 8.5% (*n* = 5 of 59), Gobiidae with 48.1% (*n* = 25 of 52) and 7.7% (*n*_impact_ = 4 of 52), Characidae with 27.3% (*n* = 9 of 33) and 24.2% (*n*_impact_ = 8 of 32), Catostomidae with 9.1% (*n* = 3 of 33) and 3.0% (*n*_impact_ = 1 of 33), Centrarchidae with 31.3% (*n* = 10 of 32) but none with reported impacts, Percidae with 12.5% (*n* = 4 of 32) and one having a reported impact (3.1%), and finally Poeciliidae with 36.7% (*n* = 11 of 30) and 3.3% (*n*_impact_ = 1 of 30) (Figure 6b).

**FIGURE 7:**
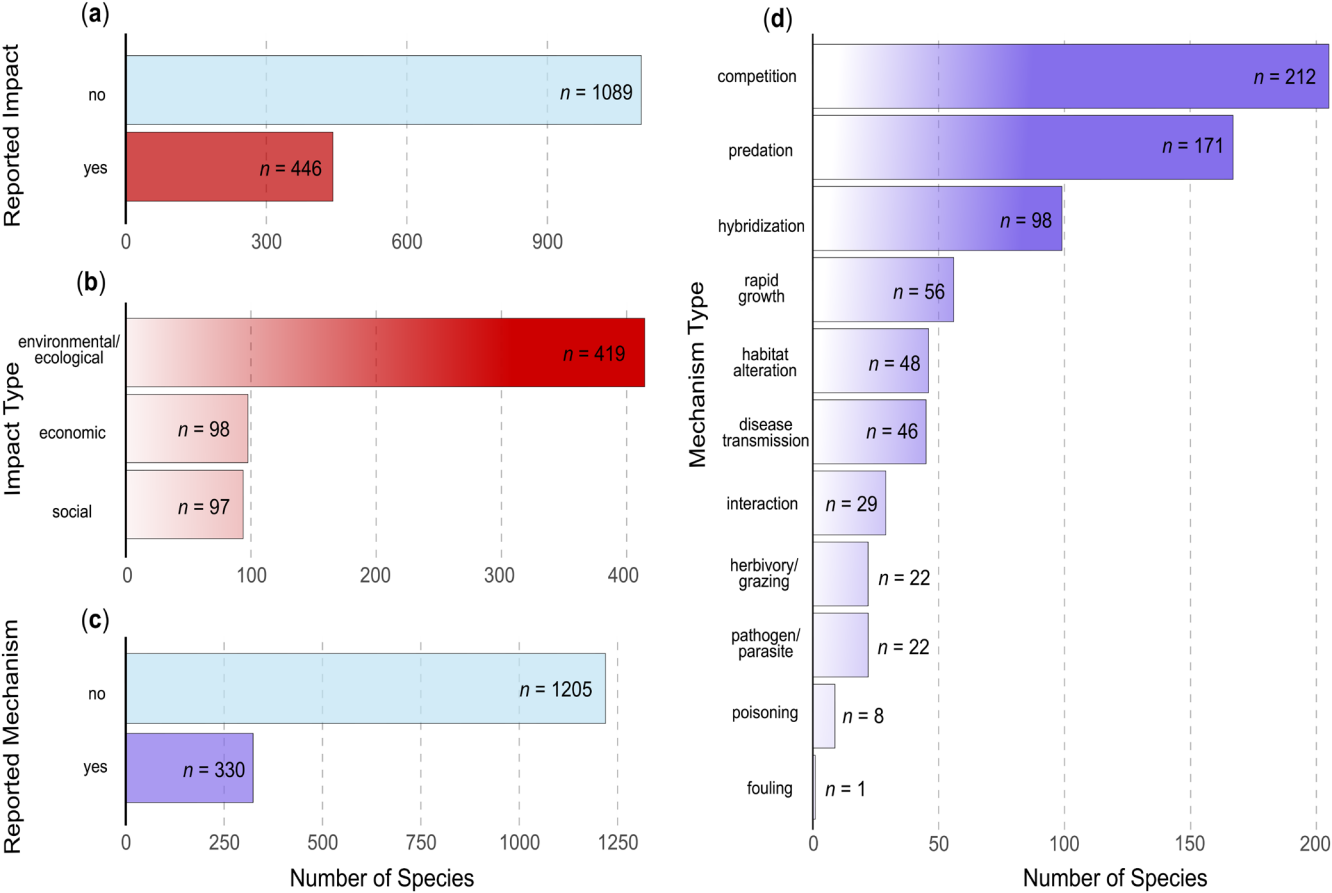
Share of established non‐native fish species with and without reported impacts (a) and the types of impacts (b), the share of species with and without reported impact mechanism (c) and the reported mechanisms (d), as well as the reported effects (e).

Through our manual search, we found impact information for 446 of the 1535 established non‐native fish species (Figure 7a), with the majority (*n* = 414) reporting ecological impacts and only *n* = 98 having known economic impacts and *n* = 97 having known social impacts (Figure 7b). Impact mechanisms were reported for *n* = 330 species (Figure 7c), with competition (*n* = 212) and predation (*n* = 171) being the main impact mechanisms (Figure 7d). Reported effects differed substantially among species (Figure 7e). Among the ten most species‐rich families, we found 25.6% (*n*_impact_ = 40 of 156) of Leuciscidae to have known impacts and 21.8% (*n*_mechanisms_ = 34) to have known impact mechanisms. For Cichlidae, 38.7% (*n*_mechanisms_ = 55 species) had reported impacts and 31.0% (*n*_mechanisms_ = 44) reported impact mechanisms, followed by Salmonidae with 23.4% (*n*_impact_ = 24) and 20.3% (*n*_mechanisms_ = 15), Cyprinidae with 37.3% (*n*_impact_ = 22) and 27.1% (*n*_mechanisms_ = 16), Gobiidae with 28.9% (*n*_impact_ = 15) and 26.9% (*n*_mechanisms_ = 14), Characidae with 27.3% (*n*_impact_ = 9) and 6.1% (*n*_mechanisms_ = 2), Catostomidae with 12.1% (*n*_impact_ = 4) and 9.1% (*n*_mechanisms_ = 3), Percidae with 34.4% (*n*_impact_ = 11) and 31.3% (*n*_mechanisms_ = 10), Centrarchidae with 62.5% (*n*_impact_ = 20) and 53.1% (*n*_mechanisms_ = 17), and Poeciliidae with 53.3% (*n*_impact_ = 16) and 40.0% (*n*_mechanisms_ = 12) (Figure 6c). A full accounting of all families and their respective number of species with reported impacts and impact mechanisms according to GRIIS and our manual search can be found in Supplement S1.

## 4 Discussion

This study presents the first comprehensive global assessment of established non‐native fish species, providing an unprecedented overview of their distribution, major introduction pathways, and associated impacts. Our findings underline the magnitude of non‐native fish introductions that have resulted in an almost global presence across diverse Earth's aquatic ecosystems. Moreover, the underlying dataset represents a crucial step forward in enhancing our understanding of non‐native fish invasions' ecological and socioeconomic consequences. As the first rigorous global compilation of established non‐native fish species, this dataset provides an invaluable foundation for invasion ecology and conservation policy. Its implications extend beyond mere documentation, as it serves as a crucial resource for future studies assessing the long‐term consequences of these introductions and their role in ecosystem transformations.

### 4.1 Fish on the Move

Our data on temporal dynamics indicated that first‐record data were available for only a proportion of established non‐native fish species, even when pooled across continents, highlighting notable taxonomic and regional gaps. While the cumulative increase in reported species was expected, the decline in new annual records in recent decades raises some crucial questions on the accuracy of previous studies as it contrasts with projections suggesting constant increases in non‐native species introductions by 2050 (Seebens et al. 2021). However, while reduced or saturated introduction rates could explain this notable discrepancy, improved management measures (e.g., restrictions in introducing fish for aquaculture, such as the EU Regulation 708/2007), or a lag in reporting and improved data documentation over time could also be contributing factors (Haubrock, Balzani, et al. 2023; Haubrock, Carneiro, et al. 2023). Nevertheless, these trends reflect only broad continental trends and do not capture country‐level variations, where introduction histories and reporting efforts are likely to differ. Regardless, the absence of first‐record data for many species highlights a critical knowledge gap that needs addressing to understand invasion dynamics and inform future management efforts. Analyses of the explanatory factors and underlying patterns, however, revealed that, despite their geographical separation across different continents, the standardized account per 10,000 km^2^, as well as temporal dynamics of reporting, exhibited minimal variation. It is also noteworthy that the countries with the highest numbers of established non‐native fish species are all highly populated and industrialized, a factor that is directly associated with significant disturbances to aquatic ecosystems, including high propagule pressure, but also physical alterations, such as damming and water diversion, as well as pollution and eutrophication (Milardi et al. 2022; Haubrock et al. 2025). It is imperative to acknowledge the potential contributions of these pathways and additional factors to the establishment of potentially harmful non‐native species, individually or synergistically.

The biogeographic origin of established non‐native fish species across different continents can provide insights into invasion pathways and underlying mechanisms of species introduction and establishment. The observed patterns suggest that many invasions originated from biogeographically and climatically similar regions, as species are more likely to establish in environments that closely match their native ecological conditions, even though some introduced intentionally and aquatic ones may deviate from this assumption (Bomford et al. 2010; Liu et al. 2020). For instance, the dominance of Palearctic species in Europe and Asia, Nearctic species in North America, and Neotropical species in South America suggests that climatic compatibility is one of numerous bottlenecks filtering introduced species, but also a species' degree of ecological niche conservatism contributes to determining invasion success (Thuiller et al. 2012). Moreover, intentionally introduced fish species are expected to be introduced where they are likely to survive (Bernery et al. 2022). Africa's unique pattern, where most established fish species originated from within the continent, further reinforces this notion, as intracontinental invasions are often facilitated by similar habitat and climate conditions, and preadapted life‐history traits that enhance establishment success (Vitule et al. 2019; Bernery et al. 2024). Additionally, historical constraints such as transport duration and infrastructure played a critical role in shaping introductions. Advances like railroads and steamships enabled the delivery of live fish—such as trout fry stocked into remote high mountain lakes—while only certain marine species could survive long sailing voyages, making these logistical barriers important ecological filters prior to modern transportation and aerial stocking (Bahls 1992; Copp et al. 2005).

The pathways by which non‐native fish species are introduced vary notably across regions, potentially reflecting differences in economic activities, historical translocations, geographic connectivity, and cultural influences, including local community pressures on policymakers to stock water bodies with economically or socially valued fish species (Gaygusuz et al. 2015; Bernery et al. 2024). Escape from confinement—primarily from aquaculture and aquarium trade—was the dominant pathway globally, followed by corridor‐mediated dispersal (particularly for marine fish) and deliberate release into nature (e.g., biological control agent, fish restocking, and fisheries enhancement). The prominence of these pathways underscores the role of human‐facilitated dispersal, with both intentional and accidental introductions shaping invasion patterns (Riera et al. 2021). Regionally, however, North America and Europe showed high numbers of escaped aquaculture and aquarium trade species, largely due to extensive fish farming industries and historical stocking programs for fisheries enhancement (Peeler et al. 2011; Tuckett et al. 2017), as well as the popularity of fishkeeping (Novák et al. 2025). The widespread presence of non‐native salmonids in temperate zones reflects this trend (Yoğurtçuoğlu et al. 2021), as do ornamental species like cichlids and poecilids in warmer regions (Maceda‐Veiga et al. 2016). In contrast, Asia, with its highly connected river networks and extensive trade routes (Brookfield 1998; Evers 2013), exhibits a stronger influence of corridor‐mediated dispersal, where non‐native fish species likely spread through interconnected waterways and modified hydrological systems. Furthermore, the high number of marine fish introduced through corridors in western Asia (Israel, Lebanon, Syria, and Türkiye) and also southern European countries is linked to the Suez Canal, which has connected the Indo‐Pacific with the Mediterranean Sea, making the latter the most invaded sea in the world (Costello et al. 2021). The significant role of intentional introductions in Asia is also evident in the prevalence of culturally and commercially valuable fish such as carps and tilapias (El‐Sayed and Fitzsimmons 2023), which have been widely introduced for food production and religious releases (Xiong et al. 2023; Yongo et al. 2023; Du et al. 2024).

In South America and Africa, non‐native fish introductions were increasingly linked to fisheries‐driven translocations and aquaculture expansion (Kang et al. 2023), although data gaps remain. The lower numbers of established non‐native species in these regions, relative to North America and Asia, may reflect lower propagule pressure and fewer historical introductions (Lockwood et al. 2005), but also less comprehensive reporting (Colautti et al. 2006) and numerous intra‐country introductions (Vitule et al. 2019). Meanwhile, Oceania, characterized by geographical isolation, exhibits a dominance of deliberate introductions, particularly for recreational fisheries (e.g., trout stocking) and biocontrol programs (Lintermans 2004).

### 4.2 The Scale of Ecological and Economic Threats

The threat presented by the 1535 established non‐native fish species reported in aquatic ecosystems across 193 countries is substantial. Their success is largely driven by the intertwined functioning effects of both high propagule pressure (due to anthropogenic activities; Johnston et al. 2017; Comte et al. 2021) and a suite of competitive advantages, including high reproductive and growth rates, as well as adaptability to human‐modified habitats—traits that often enable them to outcompete native species (Dominguez Almela et al. 2021; Nepal et al. 2024). A recent global analysis revealed that the most successful invasive freshwater fish are large‐sized, have wide diet and temperature tolerance, and high levels of parental care (Bernery et al. 2023). The impacts of non‐native fish introductions are thus diverse, spanning ecological, evolutionary, and socioeconomic dimensions, yet impacts were reported only for *n* = 80 fish species according to GRIIS in contrast to *n* = 446 species identified in our manual search. The most immediate and observable effects are the competitive exclusion or population decline due to predation of native species (Vitule et al. 2009; Tsirintanis et al. 2022). Their more efficient resource acquisition and increasing abundance can indirectly impact native species, which are simultaneously affected by habitat degradation (Tarkan et al. 2012; Błońska et al. 2016). The introduction of novel predators into ecosystems can lead to drastic shifts in population dynamics, particularly in environments where native species have not evolved defenses against such pressures (DeRoy et al. 2020). It is also important to recognize that these observed impacts may partly reflect biases, as species selected for intentional or surviving unintentional translocations often possess traits favoring survival and establishment. Thus, studies are more likely to detect impacts among those that successfully establish (i.e., a form of survivor bias; Lockwood 2021), complicating the inherent distinction between invasion risk from the outcome of past human choices and detection probabilities. A prominent example is the introduction of the European catfish ( *Silurus glanis* ) by fishermen to enhance recreational fisheries in Southern Europe, because the impacts of *S. glanis* include the extirpation of many native and non‐native fish due to its top‐predator position and ability to consume a wide range of prey (Guillerault et al. 2015, 2019; De Santis et al. 2024). Similarly, the invasion of lionfish ( *Pterois volitans* and *Pterois miles* ) in the western Atlantic and Mediterranean has led to significant declines in native fish populations and altered community structures, with lionfish reaching densities up to ten times higher than in their native range (Côté et al. 2013; Hixon et al. 2016). Ecosystem‐level changes, caused by an alteration of habitat structures, influencing sediment stability, aquatic plant growth, and hydrodynamic processes, can trigger feedback loops that amplify ecological degradation, such as increased turbidity reducing light penetration and subsequently affecting primary production (Reynolds and Aldridge 2021). The disruption of food webs further complicates ecosystem recovery, concomitantly making them more susceptible to additional invasions (Simberloff 2006; Britton 2023).

Our findings highlighted North America and Asia as the most impacted regions, likely due to aquaculture, fisheries translocations, man‐made corridors, and human‐facilitated dispersal that played key roles in shaping invasion patterns (Kang et al. 2023; Dean et al. 2024). Despite their widespread presence, impact data remain scarce, with only a third of reported non‐native species having documented ecological effects according to GRIIS, GIDIAS, and our manual search. Our manually conducted search, however, expands beyond ecological impacts, considering also economic and social impacts as well as impact mechanisms, providing a more profound understanding of the threat presented by non‐native fish species. In line with previous findings on the economic consequences of established non‐native fish introductions and taxonomic biases (Haubrock et al. 2022), the low percentage of species with available social impact information was not surprising. Yet, the lack of assessments for two thirds of established non‐native fish species indicates major knowledge gaps, limiting our understanding of their full ecological consequences, especially in the Global South (van Wilgen et al. 2022). Freshwater systems, particularly those with high endemism, are highly vulnerable to biodiversity loss (Ahmed et al. 2022). Beyond ecosystem disruption, non‐native fish invasions can impose significant economic burdens on fisheries, aquaculture, and water management (Mendoza et al. 2022; Gilles et al. 2025), highlighting the need for enhanced monitoring, comprehensive impact assessments (Ricciardi et al. 2013), and effective management (Britton et al. 2011; Giakoumi et al. 2019).

### 4.3 Research Bias and Caveats

The dataset aggregates various sources, offering a holistic picture of fish introductions across continents. However, it is also subject to several limitations that must be considered when interpreting our findings. The first and most obvious challenge is data heterogeneity (Early et al. 2016); records stem from various sources with differing methodologies, leading to inconsistencies in species identification, taxonomic updates, and spatial accuracy. Many historical records lack precise temporal data, complicating the identification of native ranges—especially in the case of translocated species (Tarkan, Kurtul, et al. 2024)—as well as the timing of introductions and their subsequent population dynamics. Additionally, there are biases in data availability, particularly in regions where non‐native species introductions have been poorly documented or where monitoring programs are underfunded or nonexistent (Tarkan, Bayçelebi, et al. 2024). Research efforts are also biased, mostly concentrated on a few high‐profile invaders (or families), which may also explain the gap in impact data. The lack of comprehensive, standardized reporting frameworks contributes to gaps in knowledge, especially in remote or politically unstable areas (Copp et al. 2005).

As human activities continue to facilitate the introduction and spread of non‐native fish, ongoing dataset updates will be crucial to maintaining its relevance. Future efforts must prioritize data standardization, improve species verification processes, and increase coverage in underrepresented regions to enhance the reliability of global assessments. The global scale of introductions highlights the interconnected nature of anthropogenic activities, including trade, aquaculture, and habitat modifications, which have facilitated the movement of species across biogeographic barriers. Yet, one of the most striking findings of this study is the pronounced research bias toward North America, particularly the United States, where data collection efforts have been extensive and long‐standing. This disparity, although in line with preceding research (Haubrock et al. 2022), skews our global understanding of non‐native fish introductions and their impacts. Many regions, particularly in Africa, South America, and parts of Eurasia, remain underrepresented, leading to potential underestimations of the true scale and consequences of fish invasions. The lack of data from these regions does not necessarily indicate fewer invasions but rather reflects limited research capacity, funding constraints, and inconsistent monitoring efforts (Pyšek et al. 2020; Stranga and Katsanevakis 2021). Without comprehensive assessments in these regions, our ability to develop a truly global understanding of invasion dynamics remains incomplete, as the underrepresentation of certain areas has significant implications for invasion ecology and policymaking. Many low and middle‐income nations experience rapid environmental changes due to urbanization, industrialization, and agricultural expansion, all of which can facilitate the spread of non‐native species (Bellard et al. 2016). However, without adequate documentation, the extent to which non‐native fish alter these ecosystems remains highly unclear (Gozlan et al. 2010).

## 5 Conclusion

The introduction and spread of non‐native fish species into aquatic ecosystems represent one of the most pressing and complex ecological challenges of our time. Mediated by human activities, such as aquaculture, fisheries support, opening corridors, and the ornamental fish trade, biological invasions can have profound and often irreversible effects on local biodiversity, ecosystem structure, and socio‐economic activities. Although significant progress has been made in invasion biology, critical knowledge gaps remain. Future research should focus on the long‐term ecological consequences of non‐native fish species, their cascading effects on food webs and ecosystem services, and their influence on ecosystem resilience under climate change. There is also a growing need for socio‐ecological studies to explore how human perceptions, cultural attitudes, and economic incentives shape the introduction and management of non‐native fish. Finally, the effectiveness of existing management strategies must also be critically assessed to ensure they are ecologically sound, economically viable, and socially acceptable.

## Author Contributions

**Phillip J. Haubrock:** conceptualization, data curation, formal analysis, investigation, methodology, visualization, writing – original draft, writing – review and editing. **Ali Serhan Tarkan:** investigation, writing – review and editing. **Irmak Kurtul:** investigation, validation, writing – review and editing. **Paride Balzani:** writing – review and editing. **Elizabeta Briski:** data curation, investigation, supervision, validation, writing – original draft, writing – review and editing. **Robert Britton:** investigation, resources, supervision, writing – original draft, writing – review and editing. **Antonín Kouba:** investigation, methodology, resources, writing – review and editing. **Dagmara Błońska:** data curation, investigation, validation, writing – review and editing. **Neil Angelo Abreo:** investigation, writing – review and editing. **Mariana Novello:** data curation, investigation, writing – review and editing. **Ana Clara Sampaio Franco:** data curation, formal analysis, investigation, validation, writing – review and editing. **Stelios Katsanevakis:** data curation, investigation, validation, writing – review and editing. **Giuseppe Castaldelli:** validation, writing – review and editing. **Ismael Soto:** data curation, formal analysis, investigation, visualization, writing – original draft, writing – review and editing.

## Conflicts of Interest

The authors declare no conflicts of interest.

## Supporting information


**Data S1:** gcb70451‐sup‐0001‐supinfo.zip.

## Data Availability

The data underlying this article has be accessed from https://zenodo.org/records/16286917 (https://doi.org/10.5281/zenodo.16286917).
