# Supplementary material for: A Global Account of Established Non‐Native Fish Species
Source: Glob Chang Biol. 2025 Aug 25;31(8):e70451. doi: 10.1111/gcb.70451 (PMC12379076; doi:10.1111/gcb.70451)
Supplement: Supplementary file 1 — Data S1: gcb70451‐sup‐0001‐supinfo.zip. [file GCB-31-e70451-s001.zip › gcb70451-sup-0001-supinfo.docx]

## **Supplementary Material:**

**Supplement 1.** Dataset

(see uploaded Excel file)

**Supplement 2.** Sensitivity analysis. Repetition of conducted analyses using the conservative subset outlined in the methods.

According to the conservative dataset, we identified 375 established non-native fish species globally. These were almost evenly contributed among marine (n = 132), freshwater (n = 115), and mixed freshwater and marine habitats (n = 128). Cichlidae were the most species rich with n = 42, followed by Salmonidae (n = 26) and Cyprinidae (n = 22). These three were followed by Gobiidae (n = 15), Leuciscidae (n = 15), Poeciliidae (n = 13), and Centrarchidae (n = 10) with ten or more species, whereas Osphronemidae (n = 9), Xenocyprinidae (n = 8), and Tetradontidae (n = 7) were the last families in the top ten, whereas all other families had five or less established non-native fish species (Figure S1).

Broken down by continent, Asia had the most established non-native fish species with n = 205 (with 12.3 species per country), followed by Europe with n = 162 species (12.3 species per country), North America with n = 104 species (9.4 species on average) and Africa with n = 102 species (7 species on average). The number of established non-native fish species were substantially lower for Oceania (n = 42; 3.9 on average) and South America (n = 21; 3.4 on average).

The country with the most established non-native fish species was the USA with 66 species, closely followed by Türkiye with 64 species and Israel with 63 species. The remaining top-10 countries globally were Italy (n = 50), Puerto Rico (n = 39), Lebanon (n = 38), the Philippines (n = 37), Cyprus (n = 35), Spain (n = 35) and finally France (n = 30) (Figure S2).

Origin-wise, the majority of reported non-native species followed a comparative pattern across continents with few regions being the main respective sources. Overall, the primary source regions of established non-native fish species were the Indomalaya, Afrotropical, Palearctic, Neotropical, and Nearctic realm, whereas other realms played only minor roles (Figure S3).

Overall, n = 45 established non-native fish species were reported as having impacts, whereas n = 215 species were reported as having no impact and n = 115 species not being listed in GRIIS. Cichlidae were the family with the most species reported as having an impact (n = 5 out of 52), followed by Cyprinidae (n = 4 out of 22), Characidae (n = 2 out of 2), Gobiidae (n = 2 out of 15), Osphreonemidae (n = 2 out of 9), Salmonidae (n = 2 out of 36), Scorpaenidae (n = 2 out of 3), Serrasalmidae (n = 2 out of 2), Siganidae (n = 2 out of 2), and finally the family Acipenseridae with n = 1 out of 4 species reported as having an impact. All other families had only one or no species with reported impacts (Figure S4).

For known pathways, escape from confinement (n = 169) was the main pathway, followed by corridor (n = 90) and release in nature (n = 48), finally followed by Transport (contaminant & stowaway) with merely n = 6 species categorized. The secondary pathways were, in descending order: aquaculture (n = 96 species), interconnected waterways/basins/seas (n = 90), ornamental purpose (n = 43), fishery in the wild (n = 39), botanical garden/zoo/aquaria (n = 17), an unknown pathway (n = 62), biological control (n = 6), farmed animals (n = 5), other transport as stowaway (n = 2), and finally food contaminant and ship/boat hull fouling with both (n = 1). In total, n = 217 species were reported as intentionally introduced, whereas only n = 96 were classified as unintentionally introduced.

In the case of all continents, the cumulative incline accelerated towards the mid or end of the 18th century (Figure S5).

**Table S1.** Top-10 most species rich families of established non-native fish species globally using the conservative dataset, broken down by number (#) and percentage (%) for Marine (M), Freshwater/Marine (FM) and Freshwater (F) species.

| **Family** | **Total # Species** | **# FM** | **% FM** | **# M** | **% M** | **# F** | **F%** |
| --- | --- | --- | --- | --- | --- | --- | --- |
| Cichlidae | 42 | 14 | 33.3 | 5 | 11.9 | 23 | 54.8 |
| Salmonidae | 26 | 20 | 76.9 | 0 | 0.0 | 6 | 23.1 |
| Cyprinidae | 22 | 12 | 54.6 | 0 | 0 | 10 | 45.6 |
| Gobiidae | 15 | 9 | 60.0 | 5 | 33.3 | 1 | 6.7 |
| Leuciscidae | 15 | 11 | 73.3 | 0 | 0 | 4 | 26.7 |
| Poeciliidae | 13 | 9 | 69.2 | 1 | 7.7 | 3 | 23.1 |
| Centrarchidae | 10 | 1 | 10.0 | 2 | 20.0 | 7 | 70.0 |
| Osphronemidae | 9 | 0 | 0.0 | 1 | 11.1 | 8 | 88.9 |
| Xenocyprinidae | 8 | 6 | 75.0 | 0 | 0 | 2 | 25.0 |
| Tetraodontidae | 7 | 0 | 0.0 | 7 | 100.0 | 0 | 0.0 |


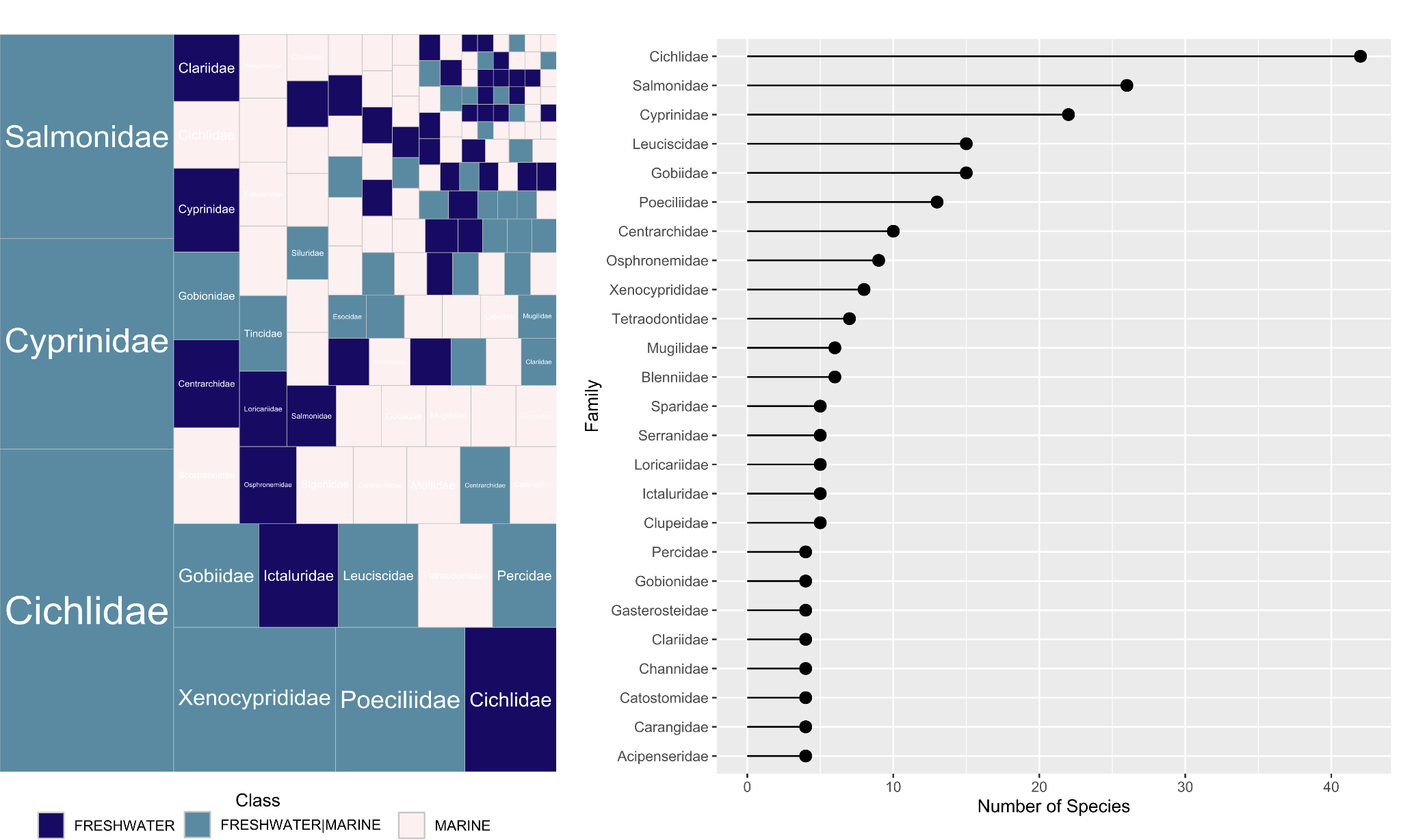


**Figure S1.** Treemap showing the distribution of total species richness among families of established non-native fish globally (a) and the top-20 most species-rich families of established non-native fish globally (b).


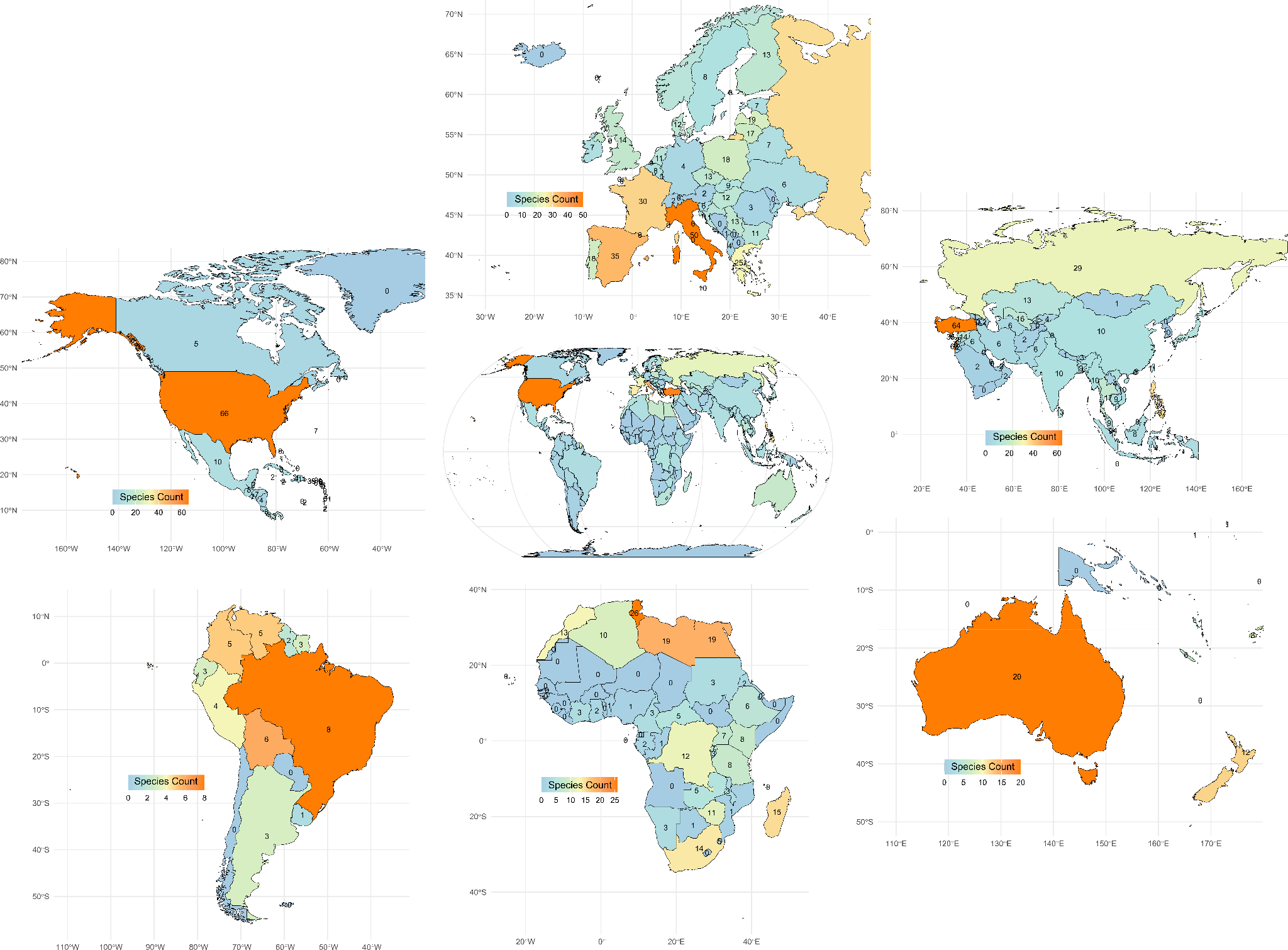


**Figure S2.** Global distribution of established non-native species broken down by continent: (**top left**) North America; (**top middle**) Europe; (**top right**) Asia; (**bottom right**) Oceania; (**bottom middle**) Africa; (**bottom left**) South America. Note that the shading for each continent’s countries is based on their own continent-specific scaling.


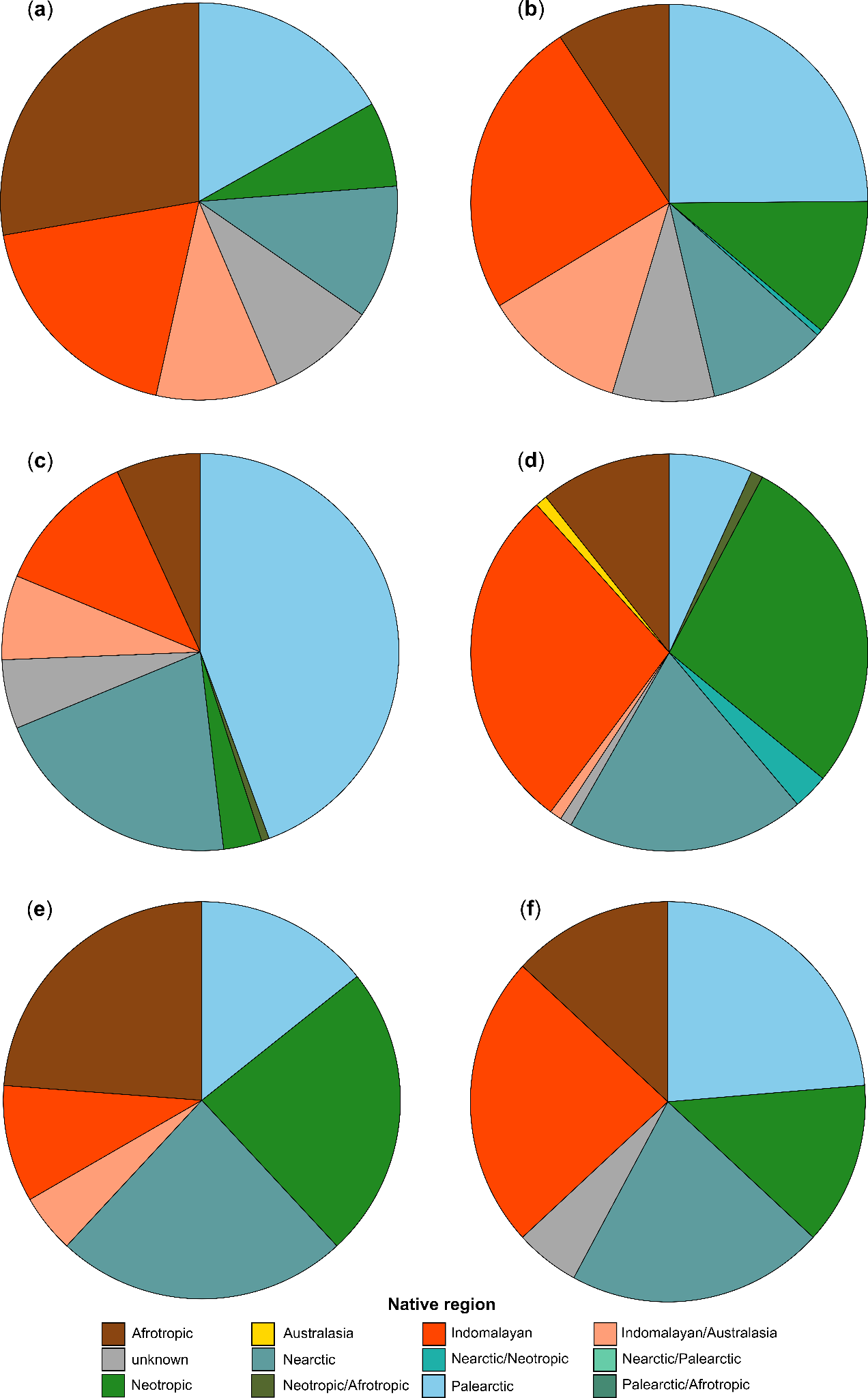


**Figure S3.** Native range of established non-native species broken down by the continent (**a**) Africa, (**b**) Asia, (**c**) Europe, (**d**) North America, (**e**) South America, and (**f**) Oceania, indicating the percentage and number for the three main contributors of established non-native fish species.


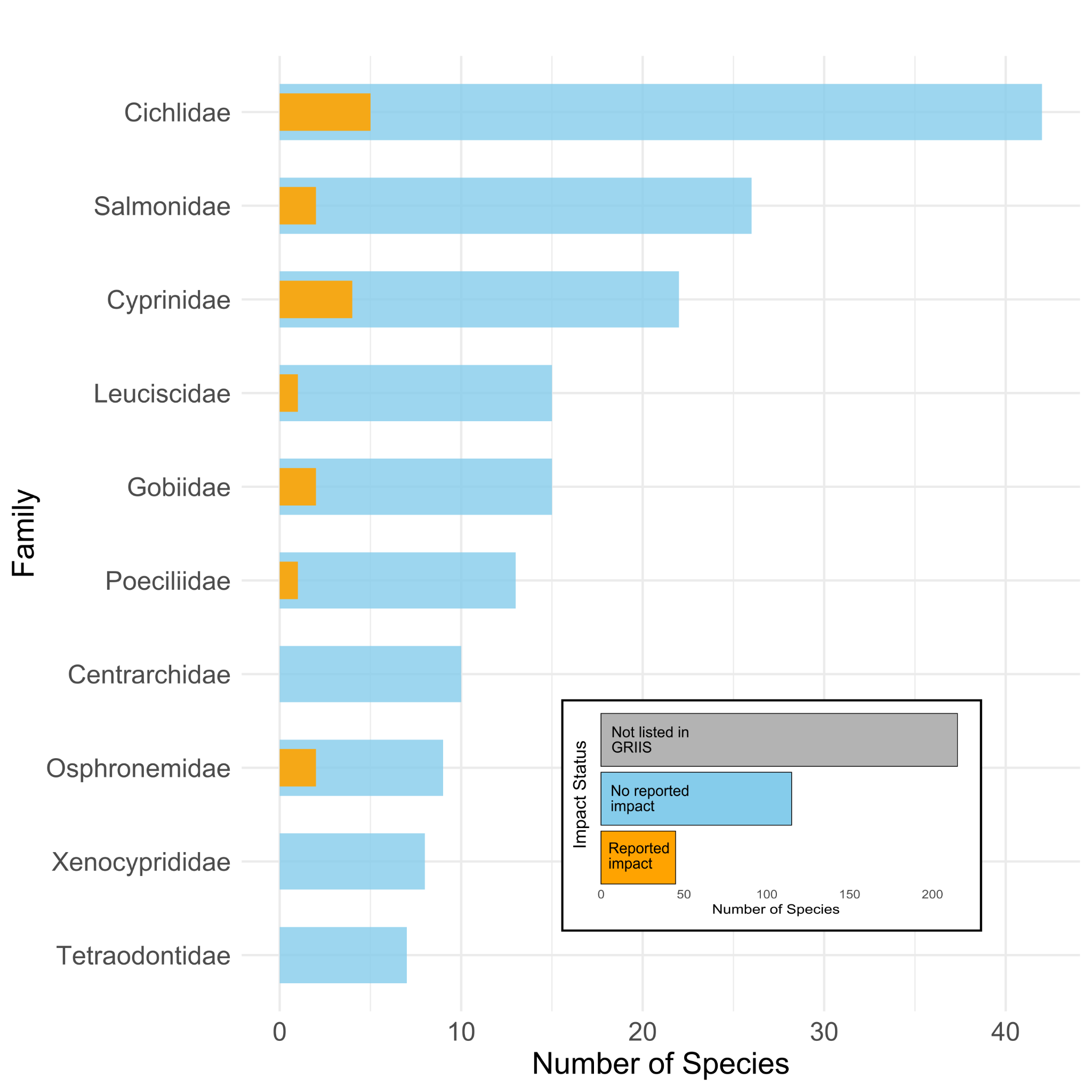


**Figure S4.** The top-10 of most species-rich families of established non-native fish species with their respective number of species for which impacts were reported in *Global Register of Introduced and Invasive Species* (GRIIS) using the conservative dataset (**a**) as well as an overview of the overall numbers of fish species which had a reported impact, had no reported impacts, or species that were missing in GRIIS (**b**).


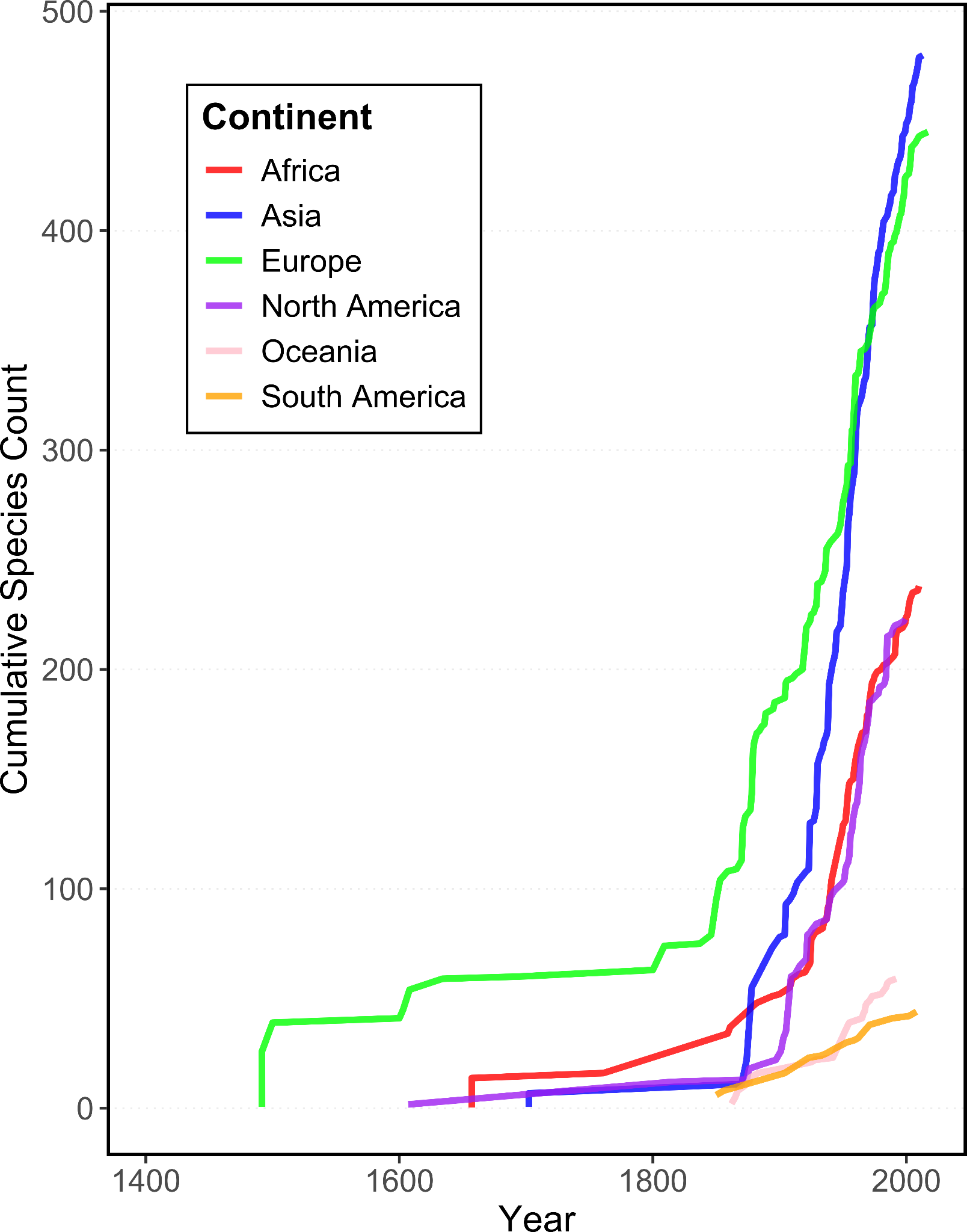


**Figure S5.** Cumulative (left y-axis; coloured area) and marginal counts (right y-axis; black bars) of established non-native fish species reported for the first time per continent (**a**: Africa; **b**: Asia; **c**: Europe; **d**: North America; **e**: South America; **f**: Oceania). The grey shaded area indicates the period before the discovery of the North American continent in 1492.

**Supplement 3.** Cumulative and marginal counts (right y-axis; black bars) of established non-native fish species reported for the first time per country (**a**: China; **b**: Indonesia; **c**: Israel; **d**: Italy; **e**: Japan; **f**: Mexico; **g**: the Philippines; **h**: Türkiye; **i**: the United States).


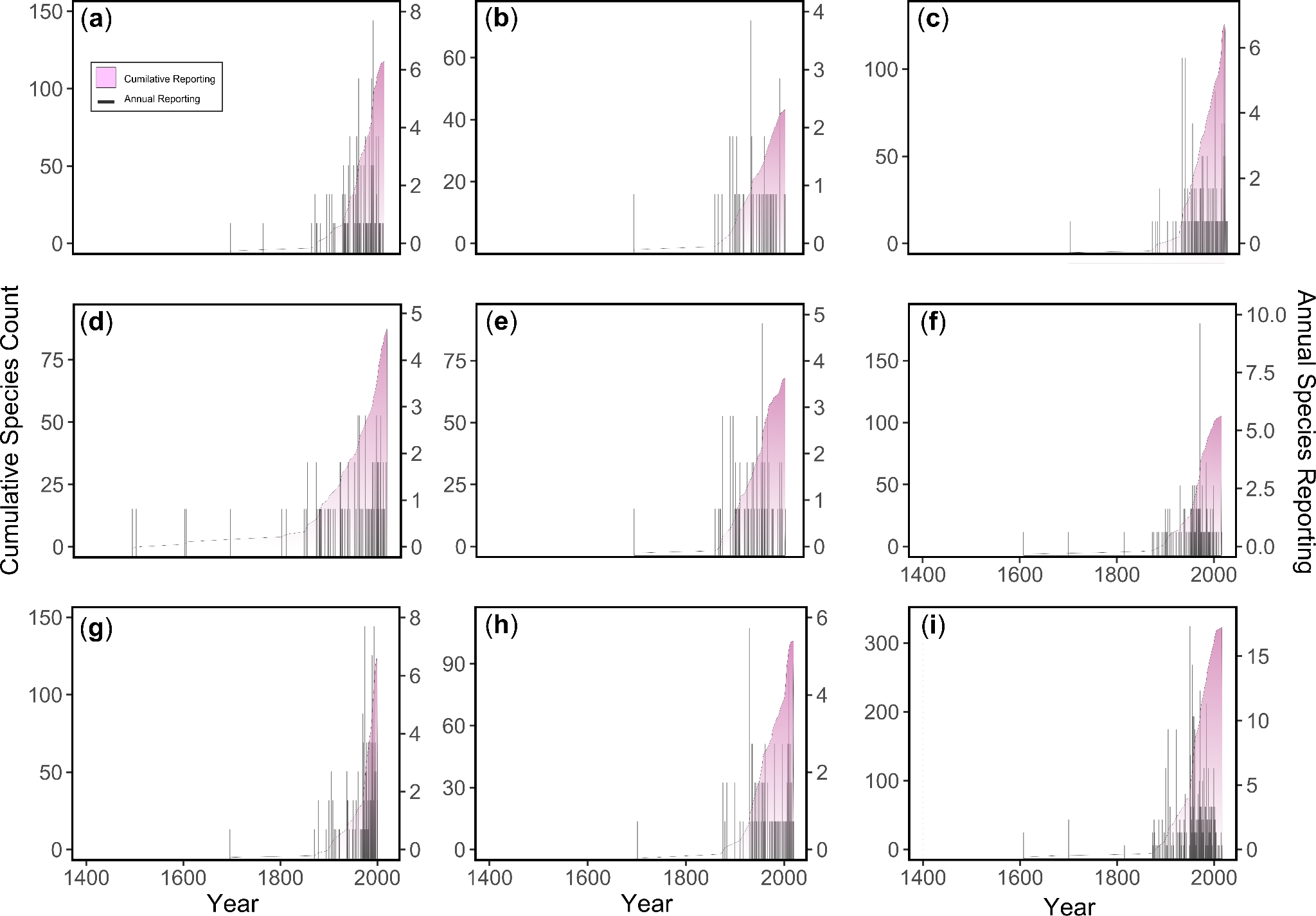


**Supplement 4.** Main pathway categories (**a**) and subcategories (**b**) available for established non-native fish species listed by Briski et al. 2024, as well as information if their introduction was intentional or unintentional according to Saul (2024) (**c**) broken down by continent.


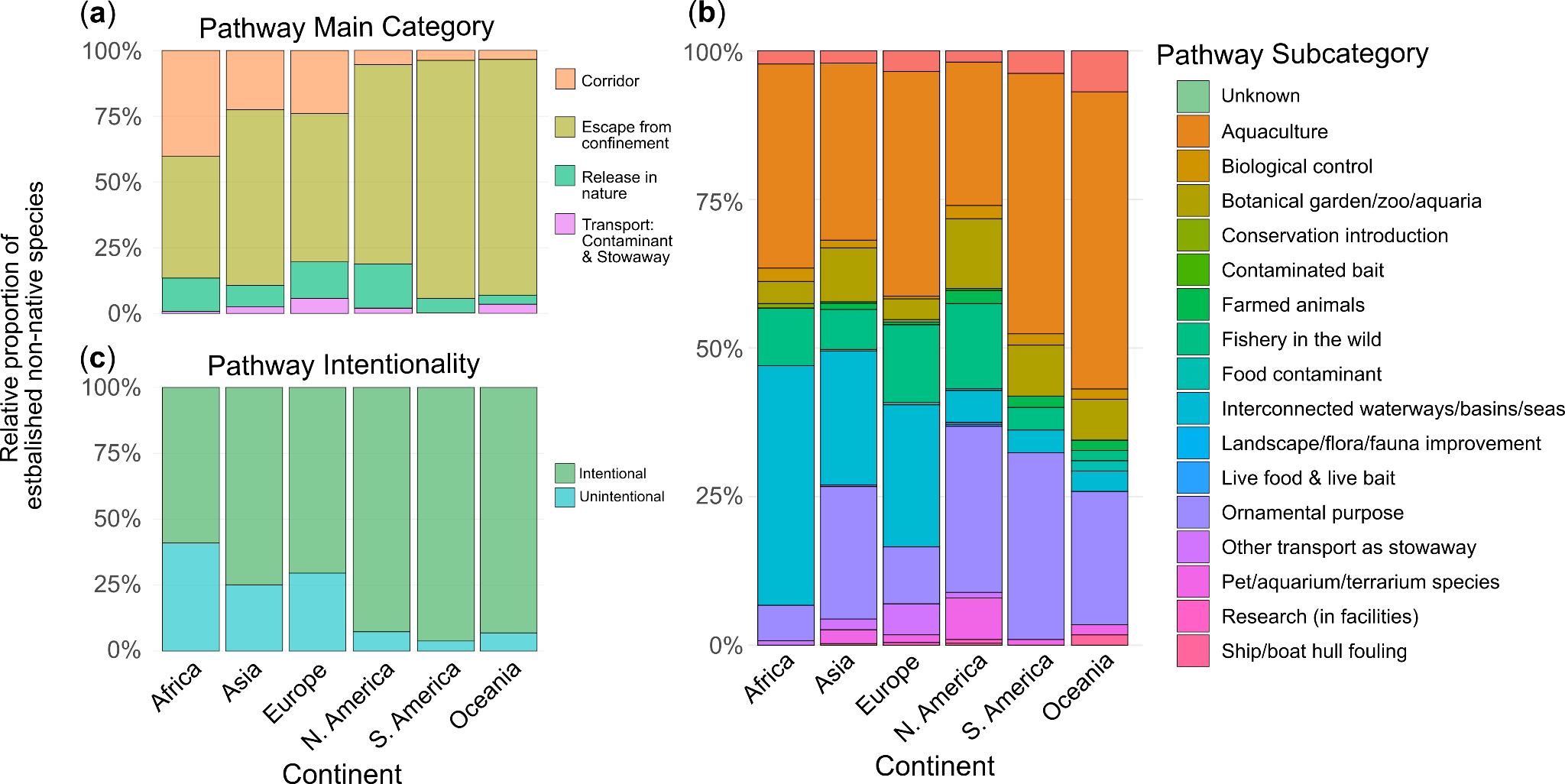


**Table S2.** Classification of pathway subcategories under their respective main pathway categories according to the Convention on Biological Diversity (CBD) framework, as used in this study. This grouping aligns with the data structure shown in Figure 5 and facilitates interpretation of introduction pathways by summarizing how specific modes of introduction (subcategories) contribute to broader pathway categories.

| **Main Pathway Category** | **Subcategories** |
| --- | --- |
| Escape from confinement | - Pet/aquarium/terrarium species - Aquaculture - Botanical gardens/zoo/aquaria - Research (in facilities) - Farmed animals |
| Corridor | - Interconnected waterways/basins/seas |
| Release in nature | - Fishery in the wild - Other intentional release - Biological control - Conservation introduction - Landscape/flora/fauna improvement |
| Transport - Contaminant & Stowaway | - Other transport as stowaway - Ballast water - Ship/boat hull fouling - Live food & live bait - Food contaminant - Contaminated bait - Contaminant nursery material |
